# *N*-(5-Chloro-1,3-thia­zol-2-yl)-2,4-difluoro­benzamide

**DOI:** 10.1107/S1600536812022477

**Published:** 2012-05-23

**Authors:** Xi-Wang Liu, Jian-Yong Li, Han Zhang, Ya-Jun Yang, Ji-Yu Zhang

**Affiliations:** a Key Laboratory of the New Animal Drug Project, Gansu Province, Key Laboratory of Veterinary Pharmaceutical Development, Ministry of Agriculture, Lanzhou Institute of Animal Science and Veterinary Pharmaceutics of CAAS, Lanzhou 730050, People’s Republic of China

## Abstract

The title compound, C_10_H_5_ClF_2_N_2_OS, was obtained by linking an amino heterocycle and a substituted benzoyl chloride. The dihedral angle between the two rings is 41.2 (2)° and the equalization of the amide C—N bond lengths reveals the existence of conjugation between the benzene ring and the thia­zole unit. In the crystal, pairs of N—H⋯N hydrogen bonds link mol­ecules into inversion dimers. Non-classical C—H⋯F and C—H⋯O hydrogen bonds stabilize the crystal structure.

## Related literature

For synthesis and the biological activity of thia­zolides, see: Ballard *et al.* (2011 ▶).
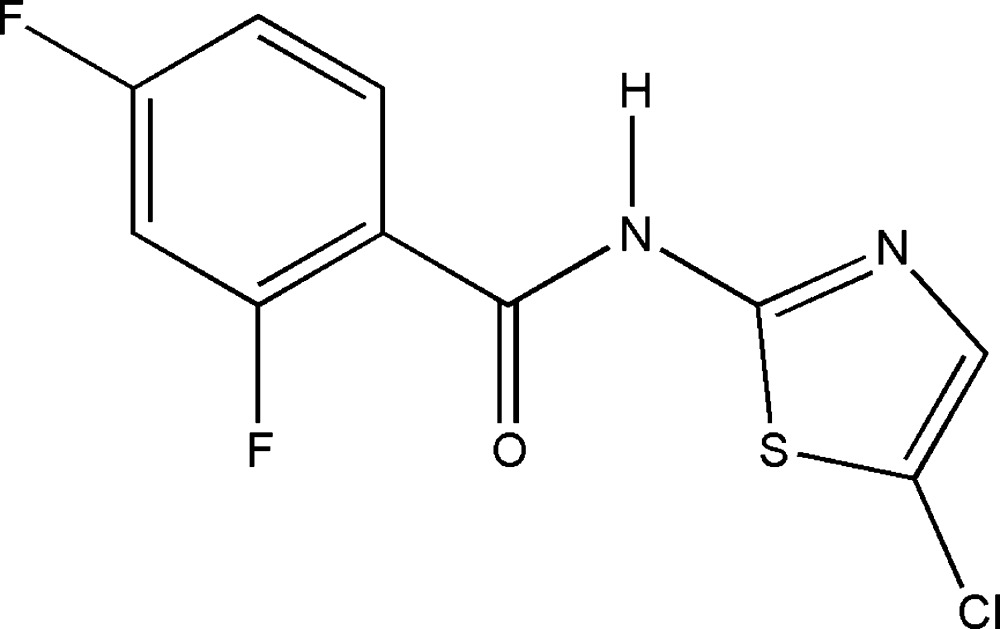


## Experimental

### 

#### Crystal data

C_10_H_5_ClF_2_N_2_OS
*M* *_r_* = 274.68
Triclinic, 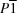

*a* = 6.929 (2) Å
*b* = 7.330 (2) Å
*c* = 12.179 (4) Å
α = 101.669 (3)°
β = 98.277 (3)°
γ = 111.796 (3)°
*V* = 545.9 (3) Å^3^
*Z* = 2
Mo *K*α radiation
μ = 0.55 mm^−1^
*T* = 296 K
0.35 × 0.33 × 0.27 mm

#### Data collection

Bruker APEXII CCD diffractometer
Absorption correction: multi-scan (*SADABS*; Sheldrick, 1996 ▶) *T*_min_ = 0.831, *T*_max_ = 0.866
3930 measured reflections
1998 independent reflections
1693 reflections with *I* > 2σ(*I*)
*R*_int_ = 0.028

#### Refinement

*R*[*F* ^2^ > 2σ(*F* ^2^)] = 0.047
*wR*(*F* ^2^) = 0.136
*S* = 1.06
1998 reflections
155 parameters
H-atom parameters constrained
Δρ_max_ = 1.25 e Å^−3^
Δρ_min_ = −0.33 e Å^−3^

### 

Data collection: *APEX2* (Bruker, 2008 ▶); cell refinement: *SAINT* (Bruker, 2008 ▶); data reduction: *SAINT*; program(s) used to solve structure: *SHELXS97* (Sheldrick, 2008 ▶); program(s) used to refine structure: *SHELXL97* (Sheldrick, 2008 ▶); molecular graphics: *SHELXTL* (Sheldrick, 2008 ▶); software used to prepare material for publication: *SHELXTL*.

## Supplementary Material

Crystal structure: contains datablock(s) I, global. DOI: 10.1107/S1600536812022477/rk2350sup1.cif

Structure factors: contains datablock(s) I. DOI: 10.1107/S1600536812022477/rk2350Isup3.hkl

Supplementary material file. DOI: 10.1107/S1600536812022477/rk2350Isup3.cml

Additional supplementary materials:  crystallographic information; 3D view; checkCIF report

## Figures and Tables

**Table 1:** Hydrogen-bond geometry (Å, °)

*D*—H⋯*A*	*D*—H	H⋯*A*	*D*⋯*A*	*D*—H⋯*A*
N1—H1⋯N2^i^	0.86	2.15	2.988 (3)	166
C4—H4⋯F2^ii^	0.93	2.38	3.127 (4)	137
C4—H4⋯O3^ii^	0.93	2.56	3.329 (4)	140

Symmetry codes: (i) 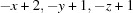; (ii) 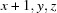.

**Fig. 1.**
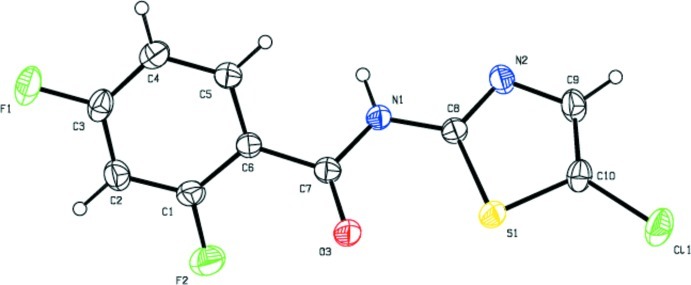
The molecular structure of title compound with the atom labels. Displacement ellipsoids are drawn at the 30% probability level. H atoms are presented as a small spheres of arbitrary radius.

## supplementary crystallographic information

### Comment

Nitazoxanide, (2-acetyloloxy-*N*-(5-nitro-2-thiazolyl)benzamide), belonged to nitrothiazole analogue, was developed as a promising compound to treat both human and animal diseases (Ballard *et al.*, 2011). In this paper, we report the synthesis and structure of the title compound, which is a derivative of nitazoxanide. The conjugation between benzene ring and thiazole moiety confirmed the existance of amide anion, which is considered to directly inhibit the *PFOR* enzyme (key enzyme of central intermidiary matabolism in anaerobic organisms). The classical intermolecular hydrogen bonds N1—H1···N2^i^ forms centrosymmetrical dimers (Table 1). The non-classical intermolecular hydrogen bonds C4—H4···F2^ii^ and C4—H4···O3^ii^ stabilize molecular packing in crystal. Symmetry codes: (i) -*x*+2, -*y*+1, -*z*+1; (ii) *x*+1, *y*, *z*.

### Experimental

The title compound was obtained according to routine method: to a solution of 5-chlorothiazol-2-amine (1 mmol) in distilled pyridine was added a equimolar amount of 2,4-difluorobenzoyl chloride with stirring. When addition was complete, the reaction mixture was allowed to stand at room temperature and stirred over night. The reaction was judged complete by *TLC* analysis. The crude product then seperated on dilution was filtered out, washed with 10% NaHCO_3_ solution, then several times with water. The dry solid was purified by chromatography to give pure compound and the crystals were obtained by recrystalization from CH_3_OH.

### Refinement

The positions of all H atoms were determined geometrically and refined using a riding model with C—H = 0.93Å, N—H = 0.86Å and *U*_iso_(H) = 1.2*U*_eq_(C, N).

### Crystal data

**Table:**

C_10_H_5_ClF_2_N_2_OS	*Z* = 2
*M**_r_* = 274.68	*F*(000) = 276
Triclinic, *P*1	*D*_x_ = 1.671 Mg m^−^^3^
Hall symbol: -P 1	Melting point = 428–429 K
*a* = 6.929 (2) Å	Mo *K*α radiation, λ = 0.71073 Å
*b* = 7.330 (2) Å	Cell parameters from 2870 reflections
*c* = 12.179 (4) Å	θ = 3.1–28.2°
α = 101.669 (3)°	µ = 0.55 mm^−^^1^
β = 98.277 (3)°	*T* = 296 K
γ = 111.796 (3)°	Block, colourless
*V* = 545.9 (3) Å^3^	0.35 × 0.33 × 0.27 mm

### Data collection

**Table:**

Bruker APEXII CCD diffractometer	1998 independent reflections
Radiation source: fine-focus sealed tube	1693 reflections with *I* > 2σ(*I*)
Graphite monochromator	*R*_int_ = 0.028
φ– and ω–scans	θ_max_ = 25.5°, θ_min_ = 3.1°
Absorption correction: multi-scan (*SADABS*; Sheldrick, 1996)	*h* = −8→8
*T*_min_ = 0.831, *T*_max_ = 0.866	*k* = −8→8
3930 measured reflections	*l* = −14→14

### Refinement

**Table:**

Refinement on *F*^2^	Secondary atom site location: difference Fourier map
Least-squares matrix: full	Hydrogen site location: inferred from neighbouring sites
*R*[*F*^2^ > 2σ(*F*^2^)] = 0.047	H-atom parameters constrained
*wR*(*F*^2^) = 0.136	*w* = 1/[σ^2^(*F*_o_^2^) + (0.0689*P*)^2^ + 0.4949*P*] where *P* = (*F*_o_^2^ + 2*F*_c_^2^)/3
*S* = 1.06	(Δ/σ)_max_ < 0.001
1998 reflections	Δρ_max_ = 1.25 e Å^−^^3^
155 parameters	Δρ_min_ = −0.33 e Å^−^^3^
0 restraints	Extinction correction: *SHELXL97* (Sheldrick, 2008), Fc^*^=kFc[1+0.001xFc^2^λ^3^/sin(2θ)]^-1/4^
Primary atom site location: structure-invariant direct methods	Extinction coefficient: 0.53 (3)

### Special details

**Table:**

Geometry. All s.u.'s (except the s.u. in the dihedral angle between two l.s. planes) are estimated using the full covariance matrix. The cell s.u.'s are taken into account individually in the estimation of s.u.'s in distances, angles and torsion angles; correlations between s.u.'s in cell parameters are only used when they are defined by crystal symmetry. An approximate (isotropic) treatment of cell s.u.'s is used for estimating s.u.'s involving l.s. planes.
Refinement. Refinement of *F*^2^ against ALL reflections. The weighted *R*-factor *wR* and goodness of fit *S* are based on *F*^2^, conventional *R*-factors *R* are based on *F*, with *F* set to zero for negative *F*^2^. The threshold expression of *F*^2^ > σ(*F*^2^) is used only for calculating *R*-factors(gt) *etc*. and is not relevant to the choice of reflections for refinement. *R*-factors based on *F*^2^ are statistically about twice as large as those based on *F*, and *R*-factors based on ALL data will be even larger.

### Fractional atomic coordinates and isotropic or equivalent isotropic displacement parameters (Å^2^)

**Table:**

	*x*	*y*	*z*	*U*_iso_*/*U*_eq_	
C1	1.0517 (5)	0.7233 (5)	0.9365 (3)	0.0438 (7)	
C2	1.2260 (5)	0.8083 (5)	1.0282 (3)	0.0494 (8)	
H2	1.2296	0.8968	1.0958	0.059*	
C3	1.3958 (5)	0.7579 (5)	1.0168 (3)	0.0467 (8)	
C4	1.3945 (5)	0.6268 (5)	0.9182 (3)	0.0464 (7)	
H4	1.5116	0.5954	0.9126	0.056*	
C5	1.2153 (5)	0.5432 (5)	0.8280 (3)	0.0413 (7)	
H5	1.2120	0.4530	0.7612	0.050*	
C6	1.0387 (4)	0.5896 (4)	0.8336 (2)	0.0358 (6)	
C7	0.8392 (5)	0.4900 (4)	0.7407 (2)	0.0390 (7)	
C8	0.6940 (4)	0.3522 (4)	0.5350 (2)	0.0365 (6)	
C9	0.5284 (5)	0.2099 (5)	0.3514 (3)	0.0505 (8)	
H9	0.5166	0.1733	0.2722	0.061*	
C10	0.3587 (5)	0.1551 (4)	0.3969 (3)	0.0447 (7)	
Cl1	0.09389 (14)	0.01753 (14)	0.32522 (8)	0.0662 (4)	
F1	1.5714 (3)	0.8434 (3)	1.10528 (18)	0.0673 (6)	
F2	0.8890 (3)	0.7783 (4)	0.9458 (2)	0.0796 (8)	
N1	0.8646 (4)	0.4602 (4)	0.62983 (19)	0.0376 (6)	
H1	0.9916	0.5106	0.6191	0.045*	
N2	0.7224 (4)	0.3246 (4)	0.4302 (2)	0.0449 (6)	
O3	0.6609 (3)	0.4314 (4)	0.75914 (18)	0.0553 (6)	
S1	0.43196 (11)	0.24360 (11)	0.54607 (6)	0.0421 (3)	

### Atomic displacement parameters (Å^2^)

**Table:**

	*U*^11^	*U*^22^	*U*^33^	*U*^12^	*U*^13^	*U*^23^
C1	0.0358 (15)	0.0438 (16)	0.0480 (17)	0.0122 (13)	0.0149 (13)	0.0095 (13)
C2	0.0483 (18)	0.0462 (17)	0.0386 (16)	0.0079 (14)	0.0089 (14)	0.0038 (13)
C3	0.0365 (15)	0.0494 (18)	0.0420 (16)	0.0035 (13)	0.0018 (12)	0.0198 (14)
C4	0.0377 (15)	0.0559 (19)	0.0504 (18)	0.0196 (14)	0.0119 (14)	0.0236 (15)
C5	0.0396 (15)	0.0447 (16)	0.0393 (15)	0.0156 (13)	0.0128 (12)	0.0123 (13)
C6	0.0329 (13)	0.0370 (14)	0.0339 (14)	0.0084 (11)	0.0107 (11)	0.0119 (11)
C7	0.0377 (15)	0.0413 (15)	0.0368 (15)	0.0135 (12)	0.0117 (12)	0.0121 (12)
C8	0.0339 (13)	0.0349 (14)	0.0372 (14)	0.0094 (11)	0.0101 (11)	0.0110 (11)
C9	0.0490 (18)	0.0455 (17)	0.0361 (16)	0.0020 (14)	0.0049 (13)	0.0052 (13)
C10	0.0414 (16)	0.0335 (15)	0.0440 (16)	0.0049 (12)	0.0006 (13)	0.0066 (12)
Cl1	0.0435 (5)	0.0572 (6)	0.0666 (6)	−0.0003 (4)	−0.0052 (4)	0.0058 (4)
F1	0.0444 (11)	0.0784 (14)	0.0528 (12)	0.0031 (10)	−0.0082 (9)	0.0204 (10)
F2	0.0495 (12)	0.0830 (16)	0.0895 (17)	0.0280 (11)	0.0141 (11)	−0.0104 (13)
N1	0.0304 (11)	0.0443 (13)	0.0336 (12)	0.0099 (10)	0.0091 (10)	0.0111 (10)
N2	0.0423 (13)	0.0449 (14)	0.0341 (13)	0.0058 (11)	0.0096 (11)	0.0062 (11)
O3	0.0349 (11)	0.0801 (17)	0.0385 (12)	0.0114 (11)	0.0119 (9)	0.0125 (11)
S1	0.0327 (4)	0.0449 (5)	0.0426 (5)	0.0098 (3)	0.0088 (3)	0.0114 (3)

### Geometric parameters (Å, º)

**Table:**

C1—F2	1.343 (4)	C7—O3	1.220 (3)
C1—C2	1.366 (4)	C7—N1	1.371 (4)
C1—C6	1.393 (4)	C8—N2	1.306 (4)
C2—C3	1.374 (5)	C8—N1	1.379 (4)
C2—H2	0.9300	C8—S1	1.729 (3)
C3—F1	1.348 (3)	C9—C10	1.334 (5)
C3—C4	1.374 (5)	C9—N2	1.378 (4)
C4—C5	1.376 (4)	C9—H9	0.9300
C4—H4	0.9300	C10—Cl1	1.719 (3)
C5—C6	1.394 (4)	C10—S1	1.730 (3)
C5—H5	0.9300	N1—H1	0.8600
C6—C7	1.480 (4)		
			
F2—C1—C2	117.5 (3)	O3—C7—N1	120.7 (3)
F2—C1—C6	119.1 (3)	O3—C7—C6	123.3 (3)
C2—C1—C6	123.4 (3)	N1—C7—C6	116.0 (2)
C1—C2—C3	117.4 (3)	N2—C8—N1	121.3 (2)
C1—C2—H2	121.3	N2—C8—S1	115.8 (2)
C3—C2—H2	121.3	N1—C8—S1	122.9 (2)
F1—C3—C4	119.0 (3)	C10—C9—N2	115.1 (3)
F1—C3—C2	118.5 (3)	C10—C9—H9	122.5
C4—C3—C2	122.5 (3)	N2—C9—H9	122.5
C3—C4—C5	118.3 (3)	C9—C10—Cl1	127.8 (3)
C3—C4—H4	120.9	C9—C10—S1	111.6 (2)
C5—C4—H4	120.9	Cl1—C10—S1	120.56 (19)
C4—C5—C6	122.0 (3)	C7—N1—C8	122.4 (2)
C4—C5—H5	119.0	C7—N1—H1	118.8
C6—C5—H5	119.0	C8—N1—H1	118.8
C1—C6—C5	116.4 (3)	C8—N2—C9	110.0 (3)
C1—C6—C7	120.8 (3)	C8—S1—C10	87.44 (14)
C5—C6—C7	122.6 (3)		
			
F2—C1—C2—C3	−177.5 (3)	C1—C6—C7—N1	145.0 (3)
C6—C1—C2—C3	0.2 (5)	C5—C6—C7—N1	−40.5 (4)
C1—C2—C3—F1	178.6 (3)	N2—C9—C10—Cl1	178.5 (2)
C1—C2—C3—C4	−0.3 (5)	N2—C9—C10—S1	−0.6 (4)
F1—C3—C4—C5	−179.1 (3)	O3—C7—N1—C8	−4.4 (4)
C2—C3—C4—C5	−0.1 (5)	C6—C7—N1—C8	173.7 (2)
C3—C4—C5—C6	0.8 (4)	N2—C8—N1—C7	−179.6 (3)
F2—C1—C6—C5	178.1 (3)	S1—C8—N1—C7	−0.1 (4)
C2—C1—C6—C5	0.4 (4)	N1—C8—N2—C9	179.1 (3)
F2—C1—C6—C7	−7.1 (4)	S1—C8—N2—C9	−0.4 (3)
C2—C1—C6—C7	175.2 (3)	C10—C9—N2—C8	0.7 (4)
C4—C5—C6—C1	−0.9 (4)	N2—C8—S1—C10	0.1 (2)
C4—C5—C6—C7	−175.6 (3)	N1—C8—S1—C10	−179.4 (3)
C1—C6—C7—O3	−37.0 (4)	C9—C10—S1—C8	0.3 (3)
C5—C6—C7—O3	137.5 (3)	Cl1—C10—S1—C8	−178.9 (2)

### Hydrogen-bond geometry (Å, º)

**Table:**

*D*—H···*A*	*D*—H	H···*A*	*D*···*A*	*D*—H···*A*
N1—H1···N2^i^	0.86	2.15	2.988 (3)	166
C4—H4···F2^ii^	0.93	2.38	3.127 (4)	137
C4—H4···O3^ii^	0.93	2.56	3.329 (4)	140

Symmetry codes: (i) −*x*+2, −*y*+1, −*z*+1; (ii) *x*+1, *y*, *z*.
